# The Utility- and Use–of Neurotechnology to Recover Consciousness: Technical and Neuroethical Considerations in Approaching the “Hard Question” of Neuroscience

**DOI:** 10.3389/fnhum.2017.00564

**Published:** 2017-11-21

**Authors:** Kathinka Evers, James J. Giordano

**Affiliations:** ^1^Centre for Research Ethics and Bioethics, Uppsala University, Uppsala, Sweden; ^2^Departments of Neurology and Biochemistry, Neuroethics Studies Program-Pellegrino Center for Clinical Bioethics, Georgetown University Medical Center, Washington, DC, United States

**Keywords:** consciousness, neurotechnology, neuroethics, neuroimaging, neurotrauma

In any attempt to recover a loss, it becomes important to assess *what* is lost, and to what extent. In clinical medicine, evaluation of loss of particular function(s) is fundamental to both diagnosis, and planning possible interventions that may be restorative. The term diagnosis is etymologically derived from the Greek *diagignoskein*, “to discern.” The root of the word, *gignṓskein* (γιγνώσκειν) means “to learn”; the implication being that one must gain knowledge about those aspects of the thing(s) that are important for it to be distinguished, identified and characterized. If, however, the focus of discernment is the clinical assessment of consciousness in a patient who is unable to respond and/or communicate through speech or overt behavior, then the act of discernment becomes complicated, given that the cardinal characteristics of consciousness are subjectivity and self-transparency, and to that extent not viable for direct objective evaluation.

Indeed, consciousness remains the elusive, proverbial “hard question” of neuroscience, and in many ways, this is exactly the point. However, it is a question and problem that cannot simply be ignored, ascribed to mere speculative explanations, or devalued. To the contrary, it must be confronted, as the discernment of consciousness is not merely a philosophical matter, but also of practical importance in, notably, neurological diagnoses, prognoses and clinical care of patients with disorders of consciousness (DoC), such as coma, minimal consciousness or persistent unresponsive states. Such considerations of diagnosis and care are not limited to the clinical milieu. They generate ethico-legal issues that conjoin other professions (e.g., law, politics) and the public in ongoing discourse and deliberation about how such patients are, or should be regarded (e.g., with respect to their capacity for sentience; ethical concerns about quality of life; legal standing, etc.,) and treated in both the short- and the long-term, inclusive of current debates about the use of brain-machine interfaces (as focal to this thematic issue), continuity and quality of care, and the justification of life-sustaining measures.

Despite considerable progress in neuroscience, the nature of consciousness (i.e., what might be viewed in Aristotelian terms as its “efficient cause”^1^) remains unknown. There are numerous theories, but at best these remain hypothetical, if not wholly notional, and often focus on some particular aspect of consciousness rather than on consciousness *per se* (Farisco et al., 2015). But if and when taken with information that posits putative brain substrates and functions involved in conscious processes (i.e., what may be more of an attempt at describing “material” or “formal” components^2^ of consciousness), such exploratory constructs may be useful to developing approaches to detect types of conscious and/or unconscious activity^3^, and in establishing possibilities methods of neurotechnological communication with patients unable to communicate through speech or overt behavior (Evers and Sigman, 2013; Farisco and Evers, 2016).

In “putting such pieces together,” we see this both as representative of “tools-to-theory-to-tools” heuristics, and as a puzzle to be solved (and suggestion of methods toward such solution), rather than an enigma simply to ponder (Gigerenzer, 1991; Wurzman and Giordano, 2009; Giordano, 2012). Toward such a goal, we offer a hypothetical equation to establish key constructs that might be methodologically engaged. Herein, let “*a/e*” stand for neural activities that are indicative of some efficient process(es) of consciousness; and “*m*” stand for material substrates (e.g., tracts, networks, and nodes) involved in particular functional domains “*d*” of these processes that are held to be important in and for clinical, ethico-legal, and social value. Given this, one methodological approach might be to assess key patterns (*P;* e.g., differential spatio-temporal arrays of tract, network and nodal engagement in the brain; behavior[s]; etc.) in specific functional domains (“*m/d*”) that are reflective of neural activity indicative of efficient processes of consciousness (“*a/e*”), such that *Pm/d* ≈*a/e* as obtained under empirically defined conditions in accordance with (the most applicably) current theoretical models of the relationship of brain function and consciousness^4^.

Still, such patterns would need to be objectively assessable. Standard clinical diagnostic criteria rely on observing patients' behaviors and characteristics in order to formulate a clinically relevant position about the type and/or level of consciousness that may be present. However, numerous studies show that, although such observations are relevant, they represent only a first step, which remains insufficient, as behaviors of patients with DoC are typically either ambiguous, or overtly absent (Farisco et al., 2015)^4^. We therefore support the view that the combined use of behavioral observations and (multiple forms of) neuroimaging could enable better conceptualization of structural and functional correlates of consciousness, which in turn could be useful to informing decisions about the kind and extent of care patients should be provided (Giordano, 2015; Evers, 2016; Farisco et al., in press)^5^. Granting that a patient with DoC may have residual consciousness to the effect that s/he experiences subjective states, and that the relevant structural and functional correlates of consciousness are identifiable, a further question arises: (how) can we adequately interpret these patients' experiences by observing brain activities (Evers and Sigman, 2013; Farisco et al., in press)?

This question is not purely technical, or philosophical. In addition to the conceptual and technical challenges to which it gives rise, it raises important neuroethico-legal and social issues focal to procedural limitations and inadequacies, liabilities inherent to the interpretation of proxy-derivative information, and inappropriate use and/or frank misuse of technology (Uttal, 2001). Acknowledging these combined challenges, we note that resolving ambiguities in neuroimaging data remains a work-in-progress. Observations of behaviors and characteristics would be instrumental to accumulating and assimilating various types of information important to further validating the viability and value of neuroimaging approaches to demonstrate structural and functional patterns of brain activity that could be signatures of consciousness^6^. Big data tools and techniques will be essential to realizing the synthesis and utility of these multi-modal and multi-leveled data; but employing computational systems and methods in these ways is not without (present and potential) problems, as well (DiEuliis and Giordano, 2016).

Such problems demand attention, and we advocate that any employment of neuroimaging should prudently assess benefit, burden and risk of the technology in specific contexts and under particular conditions (Giordano, 2017). To these ends, we call for organized efforts toward consensus in defining the patterns of neuroimaging and behavioral data that provide meaningful value in assessing DoC. This would necessitate establishment and effort of groups that are dedicated to addressing and resolving the issues that impede the clinical use of neuroimaging. Obviously, this is not a trivial task. Yet, we believe that if progress, and realistic return on the investment of time, money and expectation in neurotechnology as promoted by large scale, international efforts (e.g., the EU Human Brain Project; United States' Brain Research through Advancing Innovative Neurotechnology—BRAIN—initiative, and other, more nascent enterprises, such as the China Brain Project; Japanese Brain/MINDS Project, etc.,) are to be achieved and sustained, then efforts to translate these tools to safe and beneficial clinical applications are essential^7^. We assert that the difficulty of assessing (and treating) patients with DoC creates a viable need, and valuable opportunity to engage such technologies, which, with acknowledgement of their relative capabilities and constraints (and endeavors toward iteratively de-limiting such capabilities), could enthuse ethically sound patient-centered care.

Within an ethical perspective, patients' previously expressed values and desires are paramount in formulating best interests' standards for the use of any technique or technology in the assessment and treatment of DoC (as in any medical condition which renders the patient unable to provide consent). We have previously emphasized the importance and need for patients (and the public; i.e., as the population of potential future patients) to be knowledgeable about what technologies are available and their possible uses, allowing them to express advance directives to state whether and which neurotechnologies they, should the situation arise, would wish to be used to define—and perhaps attempt to restore—their neurological integrity (Giordano, 2015; Pascalev and Giordano, 2015).

In any case, current difficulties in correctly diagnosing DoCs should prompt new tools to be developed that decrease error or uncertainty, inclusive of assessment neurotechnologies, and more sensitive, standardized neurobehavioral metrics. But we suggest that an additional goal is not just to assess brain functions of patients with DoC, but to access them, and thereby engage the *person* in whom this brain~mind^8^ is embodied. In this context, we advocate the development and use of the ever more sophisticated forms of neurotechnological communication with DoC patients who are otherwise unable to express themselves overtly or behaviorally. To re-iterate, these approaches represent a major challenge, but the possibility and potential they hold for assessing, treating, and engaging patients with DoC are promising and ethically imperative. It is in this light—and spirit—that we view the merit of further research, innovation and discourse.

## Author contributions

All authors listed have made a substantial, direct and intellectual contribution to the work, and approved it for publication.

### Conflict of interest statement

The authors declare that the research was conducted in the absence of any commercial or financial relationships that could be construed as a potential conflict of interest.

## Footnotes

^1^Simply put, “efficient cause” refers to the thing that brings something about; in this case, “how” consciousness occurs in/from the physical substance of neural tissue(s).

^2^“Material cause” refers to the matter from which neural tissues (and, by extension, the brain) are made; “formal” cause refers to the pattern of matter and its activities that make neural tissues into a brain (that could be capable of particular functions, such as consciousness).

^3^This is important, as we believe that consciousness need not be the sole focus of care: what is currently referred to as the “unconscious” may be more complex and more morally, as well as clinically, relevant than previously imagined.

^4^This approach presumes a perspective of token physicalism, in which consciousness and other cognitive processes are “tokens” (representative, but not directly identical entities) of (putative) functions in physical structures. Thus, the goal is to iteratively lessen (or explanatorily bridge) the “token gap” between conscious/cognitive processes and functions of physical elements of the brain.

^5^Viable forms of neuroimaging that could be co-employed toward these ends include types of functional magnetic resonance imaging (fMRI), such as real-time and molecular fMRI; magnetic tractographic methods, magnetic and quantitative encephalography (MEG; qEEG); and local field recording via implanted electrodes (e.g., corticography and/or deep brain recording). For overview of these methods, (see Masdeu and Gonzalez, 2016), and/or ongoing reports in *Frontiers in Neuroscience: Neuroimaging* (https://www.frontiersin.org/journals/neurology/section/applied-neuroimaging).

^6^Such as in unresponsive wakeful state, and/or coma.

^7^Clearly, such effort(s) will require substantive financial support. Estimating costs to develop these projects, and their specific foci in elucidating a consensus registry of imaging patterns that are reflective of (states of) consciousness is difficult for a number of reasons. First, such effort will likely not be unilateral, but instead will involve multiple countries' enterprises with shared and/or contributory resources, which are funded through differing economies. Second, costs of research differ based upon settings and circumstances entailed by such projects; and third, attempts at formulating such costs require “deep dive” access into the differing costs and budgets of representative nations' projects, which is beyond the scope and focus of the present paper, but is a study in which we are currently involved, as relates to comparisons of US BRAIN initiative, EU Human Project, and proposed China Brain Project funding parameters and trajectories.

^8^Here we employ the tilde symbol (~) to denote some type of complementarity of brain and mind. Although an efficient relationship of brain and mind remains unknown, it is apparent that these entities exist and function as/in a complementarity: defined both as (a) a situation in which two or more things affect each other's qualities; and (b) the concept that the functions of two entities (or theoretical constructs), may explain particular phenomena that involve or seem to reflect both, yet each only accounts for certain aspects (e.g. - a dyad).
